# Aerobic exercise promotes the expression of ATGL and attenuates inflammation to improve hepatic steatosis via lncRNA SRA

**DOI:** 10.1038/s41598-022-09174-0

**Published:** 2022-03-30

**Authors:** Baoai Wu, Chong Xu, Yiming Tian, Yu Zeng, Feng Yan, AnPing Chen, Jinfeng Zhao, Longchang Chen

**Affiliations:** 1 Research Center for Human Movement Science, School of Physical Education, Shanxi University, Taiyuan, 030006 China; 2 Wuhan Institute of Bioengineering, Wuhan, 430415 China

**Keywords:** Diseases, Health care

## Abstract

The role of aerobic exercise in preventing and improving non-alcoholic fatty liver has been widely established. SRA is a long non-coding RNA, which has received increasing attention due to its important role in lipid metabolism. However, it is unclear whether aerobic exercise can prevent and treat hepatic lipid accumulation via SRA. The mice were randomly divided into four groups as follows, normal control group, normal aerobic exercise group, high-fat diet group (HFD), and high-fat diet plus aerobic exercise (8 weeks, 6 days/week, 18 m/min for 50 min, 6% slope) group (HAE). After 8 weeks, the mice in the HAE group showed significant improvement in hepatic steatosis. Body weight as well as blood TC, LDL-C, and liver TG levels were significantly lower in the HAE group than in the HFD group. Compared with the HFD group, the expression of SRA was markedly suppressed and the expression of ATGL was significantly increased in the HAE group. Additionally, the JNK/P38 signaling was inhibited, the pro-inflammatory factors were down-regulated, and the anti-inflammatory factor was increased. In addition to this, the same results were shown in experiments with overexpression of SRA. The results of this study provided new support for aerobic exercise to improve hepatic lipid metabolism via lncRNA.

## Introduction

Non-alcoholic fatty liver disease (NAFLD) begins with hepatic steatosis, a clinicopathological alteration characterized by the over-accumulation of lipids in hepatocytes^1,2^ Due to the high prevalence globally (approximately 25% of adults) and its serious complications, including hyperlipidemia, type 2 diabetes, insulin resistance (IR), and obesity, NAFLD has become the primary health issue^3,4^. In addition, in the occurrence and development of hepatic steatosis, disturbances in lipid metabolism are usually associated with an inflammatory state^5,6^.

Long non-coding RNAs (lncRNAs) are defined as transcripts over 200nt, lacking any coding capacity^7^, and are emerging as an important new regulator that affects a variety of biological processes and pathogenesis of metabolic diseases^8,9^. One notable lncRNA closely related to lipid metabolism is the steroid receptor RNA activator (SRA), which has been shown to affect diet-induced obesity, glucose tolerance, and hepatic steatosis^10,11^. Adipose triglyceride lipase (ATGL), a lipid droplet surface protein, is the major cytoplasmic triglycerides (TG) lipase in the process of lipolysis^12,13^. Forkhead box protein O1 (FoxO1) is a central regulator of metabolism in a variety of cell types and tissues^14^, and ATGL expression is controlled by FoxO1 as a target gene^15^. Notably, a separate study confirmed that SRA reduced ATGL expression independently of insulin signaling by inhibiting the transcriptional activity of FoxO1^16^.

The activation of the JNK/P38 MAPK signaling pathway can increase the production of hepatic inflammatory cytokines, leading to hepatic steatosis^17^. Previous studies have established that SRA modulates phosphorylation of the P38/JNK signaling pathway to regulate adipogenesis^18^. In addition, SRA inhibits the production of inflammatory factors in both in vivo and in vitro knockout experiments^18,19^. Therefore, SRA is likely to be a key potential lncRNA for improving the inflammatory response to hepatic steatosis via the MAPK signaling pathway.

Although extensive research is ongoing, the first-line of therapy is still only exercise and dietary interventions, with no approved pharmacological interventions or surgical treatments at this time. Aerobic exercise is a low-risk, non-pharmaceutical intervention that is readily available to the vast majority of the general public^20^. It effectively reduces variables associated with NAFLD, such as body weight, serum cholesterol levels, and intrahepatic fat, by inhibiting the expression of lipogenic genes^21,22^. It can also ameliorate hepatic inflammation by inhibiting pro-inflammatory mediators such as tumor necrosis factor-α (TNF-α) and interleukin-1 (IL-1)^23^. Several recent studies have reported alterations in lncRNA expression by exercise in the metabolic syndrome^24,25^, but the potential mechanisms by which aerobic exercise prevents and ameliorates lipid accumulation in hepatocytes via lncRNA remain unknown.

In this study, we examined whether aerobic exercise could affect SRA expression and how SRA could promote ATGL expression and suppress inflammation to improve hepatic steatosis in mice on a high-fat diet.

## Methods

### Ethical approval

This protocol was approved by the Scientific Research Ethics Committee of Shanxi University and China Institute for Radiation Protection. All experiments conformed to local and international guidelines for the ethical use of animals, and every effort was made to reduce the number and suffering of animals used. This study adhered to the ARRIVE guidelines. Approval Number: CIRP-IACUC-(R)2019014.

### Animals study

**Figure 1:**
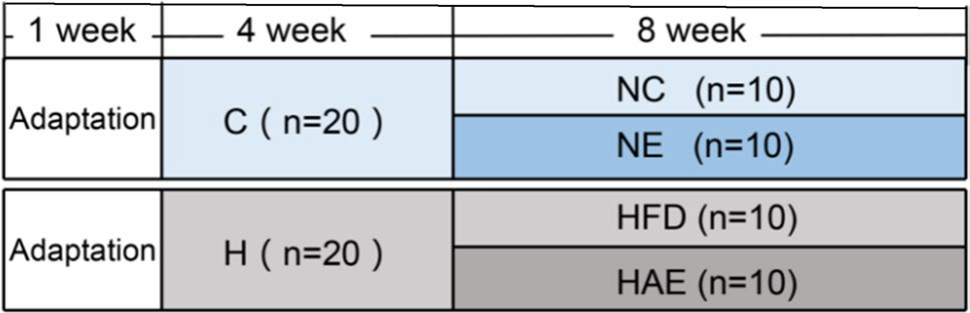
Animal groups Abbreviations: NC, normal control group; NE,normal aerobic exercise group; HFD, high-fat diet group and HAE, high-fat diet with aerobic exercise group.

Forty 8 week-old male C57BL/6J mice weighing 21.4 ± 0.92 g were purchased from Beijing Vital River Laboratory Animal Technology Biotech (Beijing, China). The animal license number is SCXK (Beijing) 2016-0006. After 1 week of adaptive feeding, the mice were randomly divided into four groups: normal control group (NC, n = 10), normal aerobic exercise group (NE, n = 10), high-fat diet (60% fat, 20% carbohydrate, 20% protein) group (HFD, n = 10)^16^, and high-fat diet with aerobic exercise group (HAE, n = 10). The energy provided (Kcal/100 g) for each diet was: Control diet: 100 g/380 kcal; High-fat: 100 g/520 kcal; The NE group received a normal diet for 4 weeks followed by aerobic exercise intervention for 8 weeks, the HFD group received a 12 week high-fat diet, and the HAE group was given aerobic exercise intervention for 8 weeks after a high-fat diet for 4 weeks. The NE group, as the exercise control group, was fed a normal diet both during adaptive feeding and exercise. The HAE group, as the high-fat exercise group, was given a high-fat diet for both the modeling and exercise periods. The animal groups are shown in Fig. 1.

To specifically overexpress lnc RNA-SRA in mice, we used a mouse lnc RNA-SRA sequenced adenovirus (Sangon Biotech, Shanghai, China). The protocol was approved by China Institute for Radiation Protection (CIRP): Approval Number CIRP-IACUC-(R)2019014. The virus was assayed in 293A cells and the virus titer was measured. Lnc RNA-SRA sequence was correct and the virus was successfully packaged at a titer of 9 × 10^11^ PFU/ml. Meanwhile, the packaging of an unrelated sequence control adenovirus was assayed and the virus was successfully packaged at a titer that met the experimental requirements. Adenoviral vectors (100 μl) were injected into the tail vein of mice for 5 weeks and divided into three groups: virus empty vector as a negative control (EV, n = 10), SRA overexpression group (SRA+, n = 10), SRA overexpression and combined with 5 weeks aerobic exercise group (SRA + AE, n = 10).

All mice were housed in standard laboratory conditions (12 h dark–light cycle, 20–26 °C, 40–60% relative humidity) at the China Institute of Radiation Protection and had free access to standard food and water. The body weight of the mice was recorded weekly. After the final treadmill exercise program was completed, the mice were fasted overnight and were anesthetized intraperitoneally with sodium pentobarbital at a dose of 80 mg/Kg, followed by spinal dislocation method of execution. The blood samples were collected in tubes containing EDTA, centrifuged at 3500 rpm at 4 °C for 10 min, and then the supernatant was transferred to new tubes and stored at − 20 °C. Mice livers were quickly removed and washed with cold phosphate-buffered saline (PBS), then part of the liver tissue was cut into the fixing solution, and the remaining liver tissues were frozen in liquid nitrogen, stored at − 80 °C until extraction.

### Exercise protocol

Aerobic exercise in mice was performed using an animal treadmill (SA101, Jiangsu Saionce Biotechnology Co., China). By referring to our previous studies, as well as the exercise protocols of Linden et al. and Kawanishi N et al. and combining them with the actual situation in our mouse experiments^26–28^. As a result, our exercise protocol was 18 m/min × 50 min, 6% incline, 6 days a week (starting at 7 pm), 8 weeks of intervention for the HAE group and 5 weeks for the SRA + AE group, with mice having a 5 min warm-up before each exercise session. The specific exercise protocol is shown in Table 1.

**Table 1:**
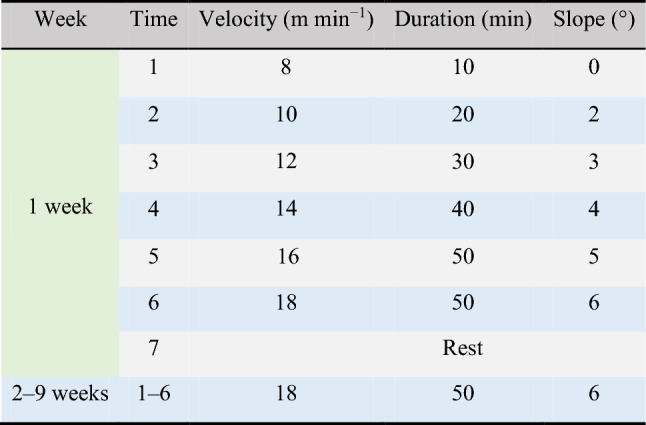
8-week-treadmill exercise protocol.

### Hepatic histology

The fresh livers were laid flat in the embedding box and immersed in the prepared 10% formalin solution; after 24–48 h the box was removed, rinsed in 50% alcohol solution, and immersed in 70% alcohol overnight. The cassette was transferred from the 70% alcohol solution to the 80% alcohol solution for 2 h. The wax was dehydrated and preserved for embedding, then sliced, spread retrieved, and dried in a warm oven. Filter hematoxylin for HE staining. The tissue was completely coated with drops of neutral gum, covered with a coverslip, and spread out to dry naturally. The degree of hepatic steatosis and lipid accumulation was observed with a light microscope (Zeiss, German).

### Blood analysis

Serum total cholesterol (TC), triglyceride (TG), LDL cholesterol (LDL-C), HDL cholesterol (HDL-C) were assayed by spectrophotometer (UV-6100s, Mapada, Shanghai, China) according to the kit’s instructions (Nanjing Jian cheng biotech, China). The content of TG and TC were measured at 510 nm, LDL-C and HDL-C were measured at 546 nm. The sensitivity of all kits: TC T-CHO Sensitivity: 0.01 mmol/l; TG T-CHO Sensitivity: 0.01 mmol/l; TG T-CHO Sensitivity: 0.01 mmol/l; LDL-C Absorbance difference ▲A of 0.180–0.280 for 2.6 mmol/l test subject; HDL-C Absorbance difference ▲A of 0.087–0.153 for 1.3 mmol/l test subject.

### Liver triglyceride analysis

Hundred mg Liver tissue was weighed at a ratio of weight (g): volume (ml) = 1:9, added to 9 times the volume of homogenising medium (saline for non-high-fat samples, anhydrous ethanol for high-fat samples), homogenised under an ice-water bath, centrifuged at 2500 rpm for 10 min and the supernatant was removed. 10 μl of 10% mouse liver homogenate was mixed and incubated at 37 °C for 10 min according to the TG assay kit (Sangon Biotech, Nanjing, China) instruction sheet. The absorbance of each tube was measured at a wavelength of 510 nm and an optical diameter of 0.5 cm. The non-high-fat samples refer to mice in the NC and NE groups mice liver samples and the high-fat samples refer to mice in the HFD and HAE mice liver samples groups. The procedure for the extraction of liver TG was the same for the NC, NE, HFD, and HAE groups and was carried out by the instructions of the Elisa kit (Sangon Biotech, Nanjing, China).

### RNA extraction and quantitative real-time PCR

The RNA was extracted by the Trizol method^29^, and the air-dried RNA was precipitated for 5–10 min after discarding the supernatant, and the precipitate was dissolved in 20 μl of DEPC water. Take 2 μl of the dissolved RNA and measure the OD260, OD280, and OD260/OD280 values by microspectrophotometer (Allsheng Instruments Co., Ltd, Hangzhou, China) to calculate the purity and concentration of RNA. The concentration of sample RNA was calculated according to the following formula based on the absorbance value: Total RNA concentration (μg/μl) = OD260 × 40 × 10^–3^. Quantitative PCR was performed on a LightCycler480 system (Roche, Switzerland) using TB Green premix Ex Taq II mix (TaKaRa, Dalian, China). mRNA expression was calculated by the 2^−△△CT^ method. Gene-specific primers were listed in Table 2, β-actin was used as an internal control. The amount of tissue used during the experiment was: 100 mg; The ratio of the weight of tissue to the volume of buffer was 1:10; Transcribed RNA: 5 μg; Amplified DNA: 25 ng.

**Table 2:** Primers for mRNA expression analysis in RT-qPCR.

Gene	Forward primer	Reverse primer
SRA	GGCGGGCTGGTGGTACTCG	GCGTCGGCTGATATCATCACATACC
ATGL	TTCACCATCCGCTTGTTGGAG	AGATGGTCACCCAATTTCCTC
1L-6	CAAGTCGGAGGCTTAATTACAC	TGCAAGTGCATCATCGTTG
TNF-α	CATCTTCTCAAAATTCGAGTGACAA	TGGGAGTAGACAAGGTACAACCC
1L-10	CCAAGCCTTATCGGAAATGA	TTTTCACAGGGGAGAAATCG
PERK	GAACCAGACGATGAGACAGAG	GGATGACACCAAGGAACCG
IRF-1a	GAGACACGGCTGGAACATCG	ACCCTGAAGGCGTTGTGGC
β-actin	GGAAAGACAACGGACAAATCAC	TACGGATCGAAACTGGCAC

SRA, long non-coding RNA steroid receptor RNA activator; ATGL, adipose triglyceride lipase; 1L-6, interleukin 6; TNF-α, tumor necrosis factor; 1L-10, interleukin 10; PERK, protein kinase R-like endoplasmic reticulum kinase; IRE-1α, inositol-requiring enzyme 1; β-actin, beta-actin.

### Western blot

Four groups of mice liver tissues were lysed in RIPA lysis buffer, protein concentration was determined by the BCA method (Beyotime, Shanghai, China). During the assay, 100 mg of liver tissue was taken and the protein concentration was measured in a ratio of 1:10 with RIPA lysis buffer. Proteins (40 μg) were separated by SDS–polyacrylamide gel electrophoresis (SDS–PAGE) and transferred onto PVDF membranes (Millipore, Billerica, USA). Soak PVDF membranes in TBST (sealing solution) containing 5% skimmed milk powder and sealed with shaking for 2 h at room temperature. Phosphorylated proteins were closed with 1% BSA. After overnight incubation with FoxO1 (1:1000, 18592-1-AP, Wuhan Sanying Biotechnology Co., Ltd.), P-FoxO1 (1:1000, ab131339, Abcam), ATGL (1:1000, 55190-1-AP, Wuhan Sanying Biotechnology Co., Ltd.), P38 (1:1000, 8690 T, CST), P-P38 (1:1000, 9211S, CST), JNK (1:1000, 24164-1-AP, Wuhan Sanying Biotechnology Co., Ltd.), P-JNK (1:1000, 24164-1-AP, Wuhan Sanying Biotechnology Co., Ltd.), P-P38 (1:1000, 9211S, CST), JNK (1:1000, 24164-1-AP, Wuhan Sanying Biotechnology Co., Ltd.), P-JNK (1:2000, Sc-6254, Santa Cruz), and β-actin (1:500, BM0627, Wuhan PhD Bioengineering Co., Ltd.) primary antibody. The membranes were washed and incubated with HRP-conjugated secondary antibody (1:5000) (Boster Biotech, Wuhan, China). Then the ECL reagent (Applygen Technologies Inc, Beijing, China) was mixed with the working solution and added dropwise to the PVDF film, the excess substrate solution was blotted off with filter paper, the film was pressed, the developer and fixer were put in sequence, and the film was rinsed.

### ATGL activity assays

The liver lysates were prepared by homogenization of tissue that was thoroughly rinsed in PBS to remove blood. ATGL activity in liver homogenates was assayed with an ELISA kit (Jiancheng Bioengineering Institute, Nanjing, China) and normalized by liver homogenates protein amount. Since (R)-bromolactone can inhibit the fraction of triglyceride hydrolase activity. The ATGL activity was obtained by calculating the difference between triglyceride hydrolase activity with and without the addition of (R)-bromolactone (25 μM). Amount of tissue used: 100 mg; the proportion between the weight of the tissue and PBS: 1:9.

### Image processing

All figures in the experiment were processed with the software Origin 2019 and Photoshop 2021 for image processing.

### Statistical analysis

All experimental data were expressed as mean ± standard deviation (mean ± SD). Statistical analysis was performed using SPSS 25.0 software (SPSS, Seattle, WA, USA), and one-way ANOVA was performed after testing for normality and chi-square. The SNK-q test was used to compare two groups and values of *P* < 0.05 were regarded as statistically significant.

## Results

### Aerobic exercise alleviated hepatic steatosis in mice

**Figure 2:**
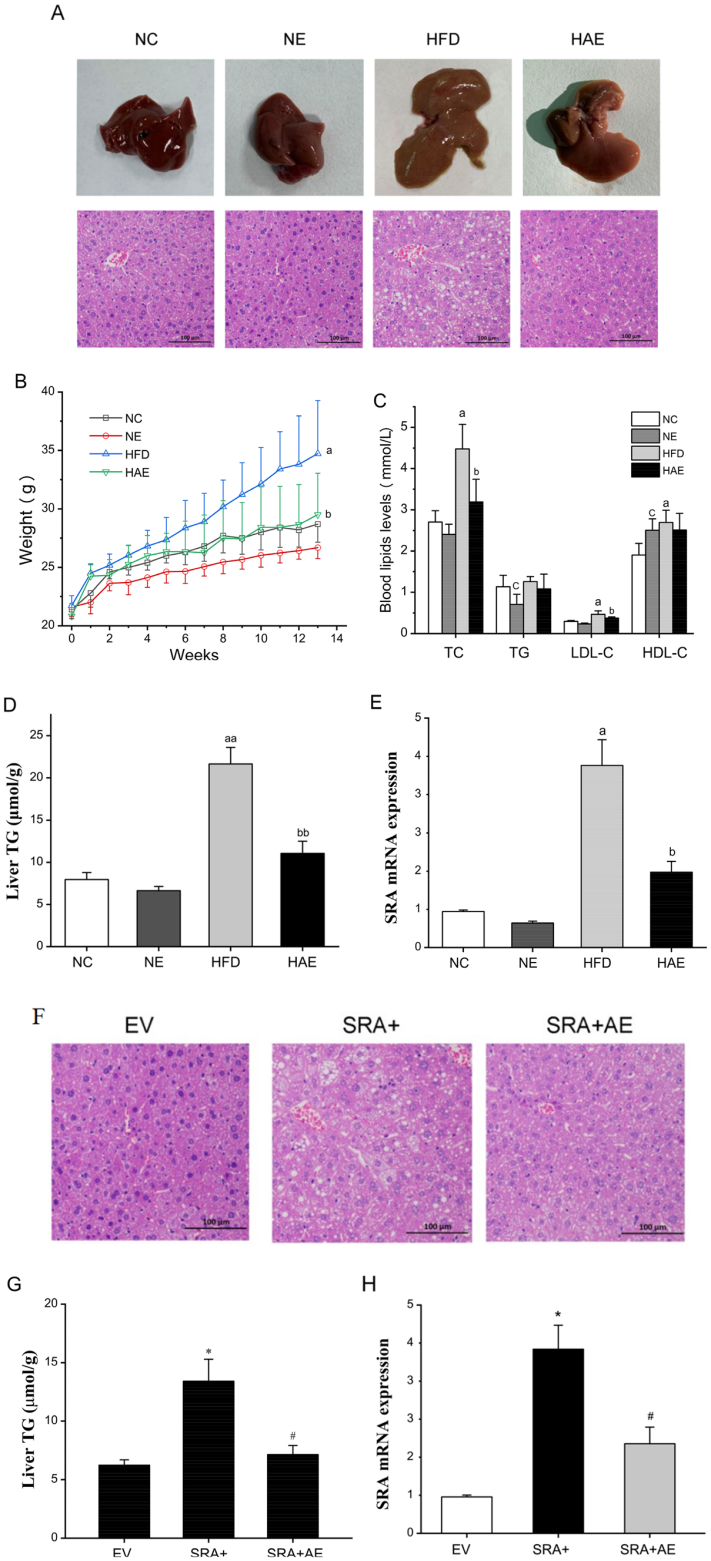
Aerobic exercise inhibits SRA expression to attenuate hepatic steatosis in mice. (**A**) Visual observation (Upper) and HE staining (Lower) to observe liver steatosis, n = 6/group. (**B**) The trend of body weight change of mice in each group, n = 10/group. (**C**) Blood lipids levels of mice in each group, n = 6/group. (**D**) Triglyceride content in mice liver, n = 6/group. a *P* < 0.05, aa *P* < 0.01 vs. NC group; b *P* < 0.05, bb *P* < 0.01 vs. HFD group; c *P* < 0.05 vs. NC group. (**E**) SRA expression in the liver of each group was measured by RT-qPCR, n = 6. (**F**) H&E stained liver sections of EV, SRA+, SRA + AE mice, n = 6/group. (**G**) Hepatic triglyceride levels in EV, SRA+, SRA + AE mice, n = 6/group. (**H**) SRA mRNA expression in the liver of each group was measured by RT-qPCR, n = 6/group. The values represent the means ± SD. *P < 0.05 vs. EV group; #*P* < 0.05 vs. SRA + group. Abbreviations: NC, normal control group; NE, normal aerobic exercise group; HFD, high-fat diet group and HAE, high-fat diet with aerobic exercise group; EV virus empty vector group; SRA+, SRA overexpression group; SRA + AE, SRA overexpression and combined with aerobic exercise group.

Morphological observations of the liver showed no abnormal changes in the livers of mice in the NC and NE groups, which were bright red with smooth envelopes and sharp margins (Fig. 2A). In HFD group mice, the liver was yellowish-brown with blunt-edged edges, swollen volume, soft texture, and oily cut surface. The liver of the HAE group returned to a state close to that of the NC group. To study the effects of aerobic exercise on liver pathology, we performed liver H&E staining analysis. As shown in the lower panel of Fig. 2A, compared with NC mice, the NE group was normal and unchanged, mice in the HFD group showed severe histopathological changes in the liver after 12 weeks of the high-fat diet, with a significant increase in the number and size of hepatic lipid droplets and accompanied by punctate necrosis of hepatocytes and infiltration of inflammatory cells. In contrast, we found that in the HAE group of mice subjected to aerobic exercise intervention, the number and size of hepatic lipid droplets were reduced, the hepatocytes were arranged neatly and hepatic steatosis was significantly improved. The body-weight of mice in the HFD group showed a rapid increase and was markedly higher than that of the NC group at the end week of the experiment (*P* < 0.05). However, from 5 to 12 weeks of aerobic exercise intervention, the body-weight of mice in the HAE group was significantly reduced compared with that in the HFD group (*P* < 0.05; Fig. 2B), the NE group showed the steadiest trend with the lowest body weight at the end of the last week of exercise but no significant difference compared to the NC group (*P* > 0.05). We also investigated the effects of aerobic exercise on blood lipid and liver triglyceride levels. After 8 weeks of aerobic exercise, compared to the HFD group, HAE group mice had significantly depressed levels of TC and LDL-C(*P* < 0.05), but not TG and HDL-C(*P* > 0.05), in their plasma (Fig. 2C). Liver TG levels were shown to be reduced by nearly half in the HAE group compared to the HFD group (*P* < 0.01; Fig. 2D). In addition, blood HDL-C and liver TG levels were significantly altered in the NE group compared to the NC group. These observations show that 8 weeks of aerobic exercise training alleviated hepatic steatosis caused by a high-fat diet in mice and improved blood lipid levels.

### Aerobic exercise inhibited SRA expression

Given the important role of SRA in liver lipids metabolism, we next studied the effects of aerobic exercise on hepatic SRA expression. The results showed that SRA expression was significantly induced in the liver of the HFD group compared to the NC group (*P* < 0.05), while the expression of SRA in the liver of HAE mice under the intervention of aerobic exercise was significantly suppressed (*P* < 0.05; Fig. 2E). In addition, to further verify the inhibitory effect of aerobic exercise on SRA, we overexpressed SRA by tail vein injection using a mouse lncRNA SRA sequenced adenovirus. Compared to the empty vector EV group, the SRA + group showed significant lipid accumulation and inflammatory cell infiltration in the liver and a significant increase in intrahepatic TG levels (*P* < 0.05, Fig. 2F, G), whereas the SRA + AE group returned to normal liver lipid accumulation and a significant decrease in TG levels after 5 weeks of aerobic exercise intervention (*P* < 0.05). Furthermore, the findings of RT-qPCR analysis showed that SRA expression was significantly increased in the SRA + group and significantly suppressed in the SRA + AE group(*P* < 0.05; Fig. 2H). These data indicate that aerobic exercise plays an important role in improving hepatic steatosis by inhibiting SRA expression.

### Aerobic exercise upregulated the hepatic ATGL expression as well as activity via SRA

**Figure 3:**
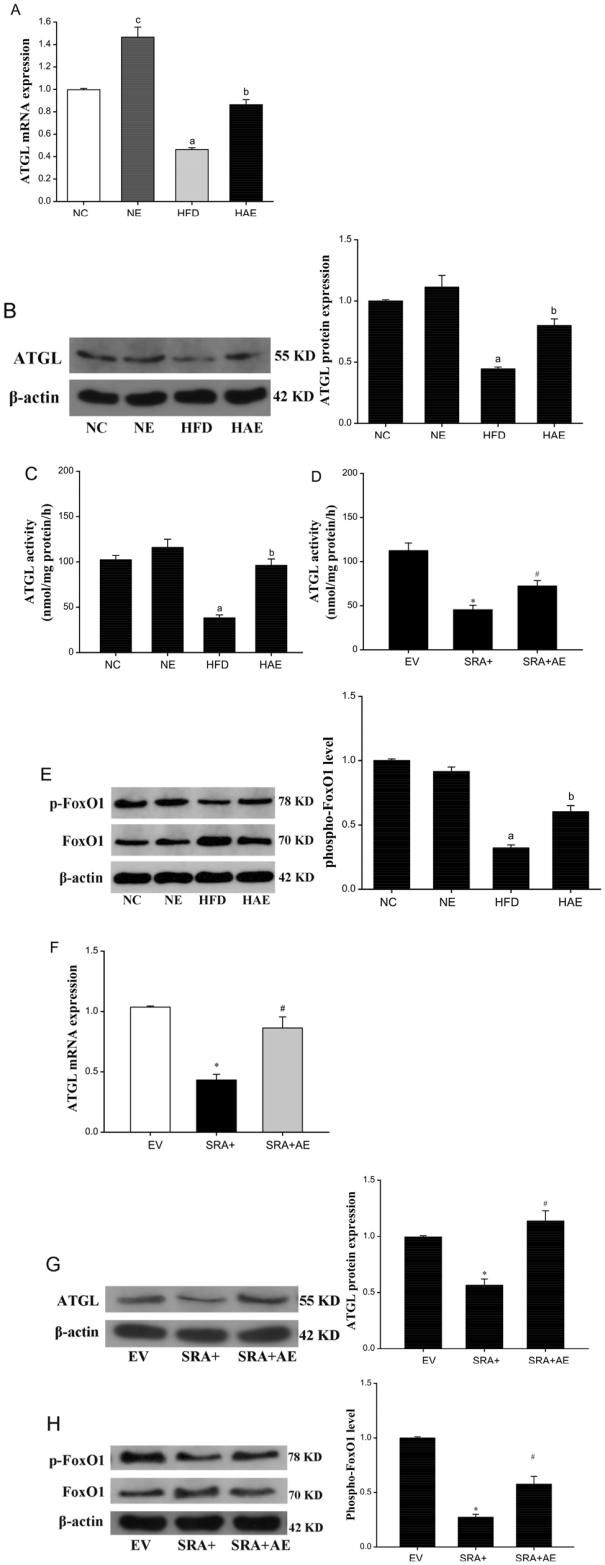
Aerobic exercise upregulates the hepatic ATGL levels via SRA. (**A**) The ATGL mRNA levels in livers of mice were measured by RT-qPCR, n = 6/group. (**B**) Protein levels of ATGL were determined by western blot. (**C**) The expression of ATGL activity in mice liver. a *P* < 0.05 vs. NC group; b *P* < 0.05 vs. HFD group. (**D**) The expression of ATGL activity in mice liver. *P < 0.05 vs. EV group; #*P* < 0.05 vs. SRA + group. (**E**) The protein expression and phosphorylation levels of FoxO1 in mice liver assessed by western blot. a *P* < 0.05 vs. NC group; b *P* < 0.05 vs. HFD group; c *P* < 0.05 vs. NC group. (F) Hepatic ATGL mRNA levels in EV, SRA+, SRA + AE groups of mice, n = 6. (**G**) Protein levels of ATGL were determined by western blot. (**H**) The protein expression and phosphorylation levels of FoxO1 in mice liver assessed by western blot. The values represent the means ± SD. *P < 0.05 vs. EV group, #*P* < 0.05 vs. SRA + group. a *P* < 0.05 vs. NC group; b *P* < 0.05 vs. HFD group; *P < 0.05 vs. EV group; #*P* < 0.05 vs. SRA + group.Abbreviations: NC, normal control group; NE, normal aerobic exercise group; HFD, high-fat diet group and HAE, high-fat diet with aerobic exercise group; EV virus empty vector group; SRA+, SRA overexpression group; SRA + AE, SRA overexpression and combined with the aerobic exercise group.

To determine whether the marked reduction in lipids observed in the livers of mice in the HAE and SRA + groups was the result of increased lipolysis, we compared the expression and activity of ATGL. After 12 weeks of high-fat diet feeding in the HFD group, the ATGL mRNA and protein levels in the liver were significantly reduced, compared with the NC group (*P* < 0.05), and aerobic exercise markedly increased the levels of liver ATGL in the HAE group mouse livers (Fig. 3A, B). The enzymatic activity of ATGL showed the same trend (Fig. 3C, B). SRA is known to inhibit FoxO1 transcriptional activity independently of insulin signaling^16^. To understand the mechanism of how aerobic exercise regulates ATGL expression through SRA, we next assessed the expression and phosphorylation levels of FoxO1, a well-known transcription factor that positively regulates ATGL^30^. The analysis of FoxO1 showed that a high-fat diet inhibited phosphorylation in the liver of mice(*P* < 0.05; Fig. 3E), which may be closely related to IR^31^, and its phosphorylation was significantly up-regulated in the HAE group (*P* < 0.05). Furthermore, the SRA + AE group also showed a significant upregulation of ATGL expression as well as FoxO1 phosphorylation levels in response to the aerobic exercise intervention (*P* < 0.05; Fig. 3F–H). These results together suggest that aerobic exercise inhibits FoxO1 phosphorylation via SRA to upregulate hepatic ATGL expression as well as activity to promote lipolysis, thereby reducing intrahepatic TG levels.

### Aerobic exercise improved inflammation through SRA

**Figure 4:**
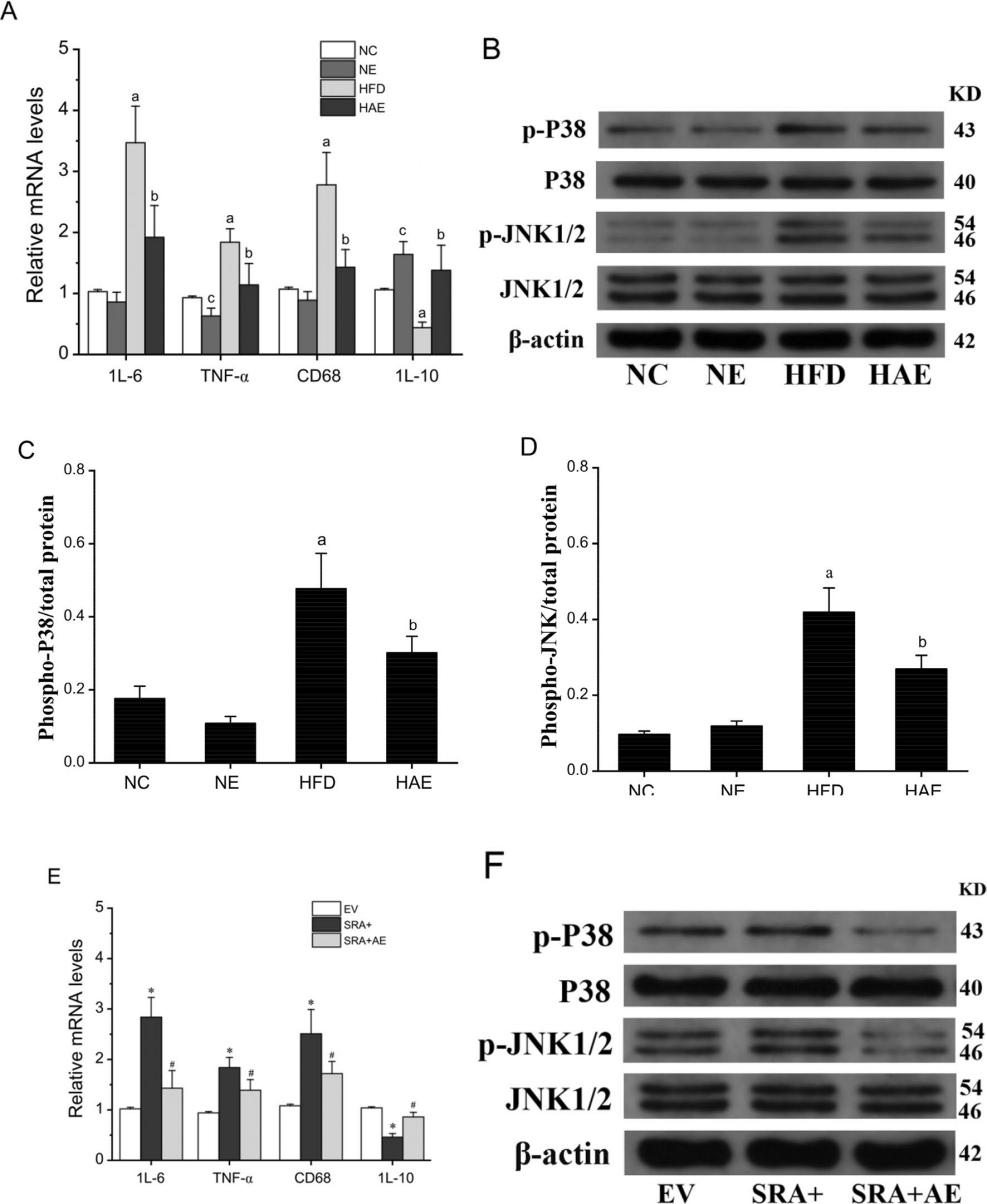

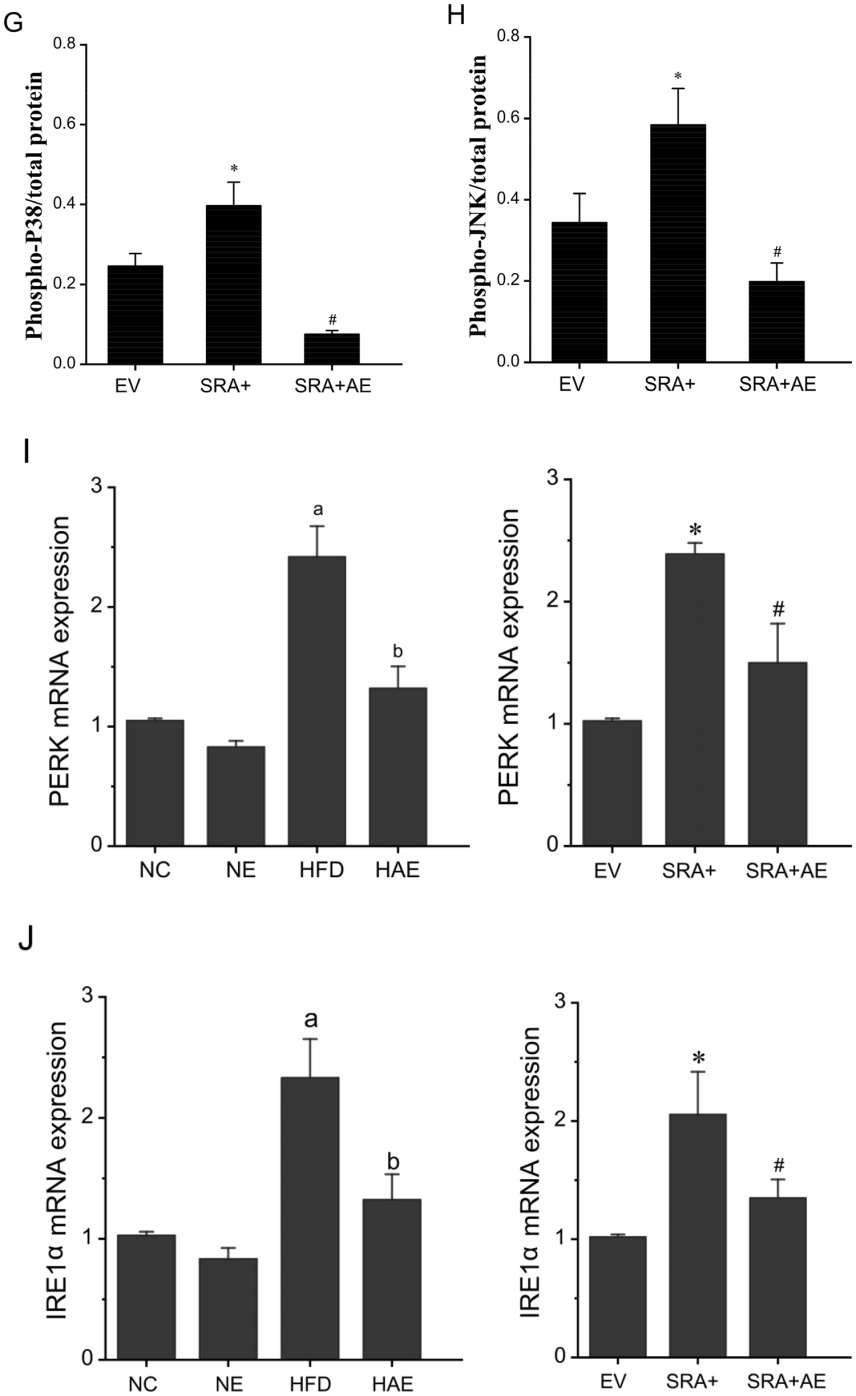
Aerobic exercise improves P38/JNK-mediated inflammation through SRA. (**A**) The mRNA levels of inflammatory factors in mice liver assessed by RT-qPCR, n = 6/group. (**B**) Western blot analysis of protein expression and phosphorylation of P38 and JNK. (**C**) The phosphorylated P38/total protein levels in the liver of each group of mice. (**D**) The Phosphorylated JNK/total protein levels in the liver of each group of mice. a *P* < 0.05 vs. NC group; b *P* < 0.05 vs. HFD group; c *P* < 0.05 vs. NC group. (**E**) The mRNA levels of inflammatory factors in the liver of EV, SRA+, SRA + AE mice were assessed by RT-qPCR, n = 6/group. (**F**) Western blot analysis of protein expression and phosphorylation of P38 and JNK in EV, SRA+, SRA + AE mice. (**G**) The phosphorylated P38/total protein levels in the liver of each group of mice. (**H**) The Phosphorylated JNK/total protein levels in the liver of each group of mice. (I) The expression of PERK activity in mice liver, n = 6/group. (**J**) The expression of IRE1a activity in mice liver, n = 6/group. a *P* < 0.05 vs. NC group; b *P* < 0.05 vs. HFD group; *P < 0.05 vs. EV group; #*P* < 0.05 vs. SRA + group. The values represent the means ± SD. Abbreviations: NC, normal control group; NE, normal aerobic exercise group; HFD, high-fat diet group and HAE, high-fat diet with aerobic exercise group; EV virus empty vector group; SRA+, SRA overexpression group; SRA + AE, SRA overexpression and combined with the aerobic exercise group.

SRA shows profound regulation of P38/JNK MAPK signaling, which is closely related to inflammation and metabolic disorders^19,32^. This relationship prompted us to investigate whether the P38/JNK pathway is involved in SRA regulated hepatic steatosis and its related pathology. As expected, we determined that aerobic exercise reduced liver mRNA levels of pro-inflammatory factors including IL-6, TNF-α, CD68, and increased levels of the anti-inflammatory factor IL-10 in mice (*P* < 0.05; Fig. 4A). Compared with mice under normal conditions, the phosphorylation levels of P38 and JNK1/2 were significantly increased in liver samples from the HFD group (*P* < 0.05; Fig. 4B–D). After aerobic exercise intervention, the phosphorylation level in the liver of HAE group mice was significantly reduced (*P* < 0.05). In addition, the pro-inflammatory markers IL-6, CD68, and TNF-α were significantly increased and the anti-inflammatory marker IL-10 was greatly decreased and JNK/P38 phosphorylation levels were increased in the SRA overexpressing mice (Fig. 4E–H). In contrast, in the SRA + AE group, the levels of pro-inflammatory IL-6, CD68, and TNF-α were obviously suppressed and the levels of anti-inflammatory IL-10 were markedly increased and the levels of JNK/P38 phosphorylation were downregulated after 5 weeks of aerobic exercise. In addition, the important influence of endoplasmic reticulum stress (ERS) on hepatic steatosis was taken into account. Here we also analyzed the expression of two signaling molecules, protein kinase-like endoplasmic reticulum kinase (PERK) and endoplasmic reticulum transmembrane kinase 1 alpha (IRE1α), in ERS as well as in inflammation. The results showed that both PERK and IRE1α mRNA expression levels showed a significant increase following a high-fat diet and overexpression of SRA, while their levels decreased significantly under the exercise intervention (Fig. 4I,J). Based on these findings, we infer that aerobic exercise suppresses the expression of SRA in hepatic steatosis to improve inflammation and is closely related to the JNK/P38 pathway and the ERS conducted by IRE1α and PERK.

## Discussion

NAFLD is one of the most common chronic liver diseases and its pathogenesis involves abnormal activation of hepatic lipogenesis and inflammation production^3,33^. However, the regulatory networks that regulate the physiological and pathological activation of hepatic lipogenic programs are not fully understood. Recently, increasing numbers of studies have revealed the importance of lncRNAs in the regulation of diverse biological processes and have been shown to play an important role in lipid metabolism^34,35^. In this study, we found that aerobic exercise suppressed the expression of SRA in the liver of mice, thereby improving hepatic steatosis (Fig. 1). The decrease in intrahepatic lipid aggregation may be due to increased expression of ATGL, which promotes the breakdown of TG.

FoxO1 acts as a transcription factor that directly regulates ATGL expression^30^. Phosphorylated FoxO1, which is regulated by insulin, binds to the chaperone 14-3-3 protein for transport from the nucleus to the cytoplasm and downregulates transcriptional activity to suppress target gene expression^31^. Phosphorylation of FoxO1 and expression of ATGL were significantly reduced in mouse liver under a high-fat diet. This is in contrast to the reduced phosphorylation of FoxO1 remaining in the nucleus increasing its effect on promoting ATGL expression. In addition, previous studies have shown that exercise training markedly increases phosphorylation of Foxo1 in the liver^36^. As in previous studies, our results suggest a significant correlation between aerobic exercise affecting Foxo1 phosphorylation and the suppression of SRA expression. It is speculated that SRA alters the transcriptional activity of FoxO1 to decrease ATGL expression, which may be unrelated to the established insulin signaling pathway that promotes phosphorylation of FoxO1 to suppress ATGL expression. These need to be further validated by subsequent studies.

Chronic inflammation is an important factor in hepatic steatosis^17^. The deficiency of SRA reduced the expression of inflammatory genes in vivo and in vitro^19,32^. Our results showed that aerobic exercise markedly inhibited SRA and reduced the expression of pro-inflammatory factor genes, while the P38/JNK signaling pathway was also found to show the same significant alterations. Recent studies confirm that endoplasmic reticulum stress is strongly associated with the development of NAFLD. PERK and IRE1α play an important role in linking endoplasmic reticulum stress to inflammatory responses. Exercise not only enhances the tolerance of the liver to ERS but also prevents the malignant development of steatosis due to excessive ERS^37^. For example, long-term aerobic exercise suppressed the expression of IRE-1α and PERK in the liver and exerted beneficial effects on intrahepatic metabolism^38^; aerobic swimming exercise reduced the expression of PERK and IRE-1α in the liver of rats and inhibited the formation of nonalcoholic fatty liver disease^39^. The results showed that both PERK and IRE1α mRNA expression levels showed a significant increase following a high-fat diet and overexpression of SRA, while their levels decreased significantly under the exercise intervention (Fig. 4I, J). The above suggests that aerobic exercise inhibits SRA expression in hepatic steatosis to ameliorate inflammation in close association with the JNK/P38 pathway and the ERS of IRE1α and PERK transduction. It also suggests a possible close link between SRA and established signaling and metabolic networks.

It is well established that many lncRNAs associated with lipid metabolism are involved in transcriptional regulation, acting through specific protein-binding partners. For instance, lncRNA Blnc1 is a core component of the LXR/SREBP1c protein pathway that is required for lipogenic induction^40^; lncRNA LSTR binds to the TDP-43 protein, resulting in a decrease in Cyp8b1 gene expression and substantial changes in bile acid composition^35^. Previous studies have indicated that SRA exerts its diverse biological functions and roles in the development of cancer and other diseases by interacting with the protein chaperones in the activation and inhibition complexes. For example, SRA regulates NR transcriptional activity by directly or indirectly interacting with NR^11^. SRA also forms complexes with core regulators of NRs and TFs, such as SRC-1, P68, PUS1/3, components of RISC complex proteins (PACT, TRBP, Dicer, and Argonaut2), and inhibitory core regulatory proteins SHARP and SLIRP^41,42^. Here, although aerobic exercise has been shown to enhance the transcriptional activity of FoxO1 through SRA to regulate ATGL expression in the liver, how SRA binds to its corepressor to promote this level of change needs to be further investigated and confirmed.

Aerobic exercise is not only for weight loss but also for improving body adaptability and function. The data presented here show that aerobic exercise successfully improved hepatic steatosis in mice fed with a high-fat diet, it also significantly improved body weight and blood lipid levels. Our previous studies found that aerobic exercise reduced the expression of PCSK9, an important gene for cholesterol metabolism^43^. Recent studies have confirmed that exercise affects the level of lncRNA in the body. For example, the GW29-e0223 LncRNA gene chip analyzes the effect of simultaneous exercise on aortic insulin resistance^24^; exercise can reduce insulin resistance in type 2 diabetes by mediating the lncRNA MALAT1/microRNA-382-3p/ resistin axis^25^. These studies suggest that exercise can exert multiple physiological modulatory effects on related metabolic diseases through LncRNAs, but the deeper mechanisms remain unclear. Therefore, exploring the mechanisms of lncRNA-related diseases and combining them with exercise to provide new therapeutic strategies would be an area of interest.

Youn Ju Kim et al. demonstrated that although 8 weeks of aerobic exercise significantly increased the diet of mice in the high-fat exercise group, the experimental results showed that body weight, liver triglyceride content, and total serum cholesterol were still significantly reduced after the exercise intervention, which contributed to the improvement of hepatic steatosis^44^. The same results were shown in the study performed by Emmanuel Denou et al.^45^. Furthermore, it was also found that swimming exercise intervention in mice with NAFLD did not produce differences in their dietary intake compared to the non-exercise group, and that body weight, liver triglyceride levels, and H&E staining results in mice after exercise intervention indicated that exercise could be very beneficial in ameliorating NAFLD^46^. By reviewing the extensive literature and comparing relevant studies, the exercise intervention in high-fat-fed mice showed a reduction in body weight and a significant improvement in hepatic steatosis, whether the diet was increased or remained unchanged. The lack of monitoring of the dietary intake of the mice was a shortcoming of our experimental design, but based on these results, we have reason to believe that the weight loss in the hepatic steatosis mice was caused by exercise. In future experimental designs, we will definitely consider the diet of the mice as a factor and thus make the experimental design better.

**Figure 5:**
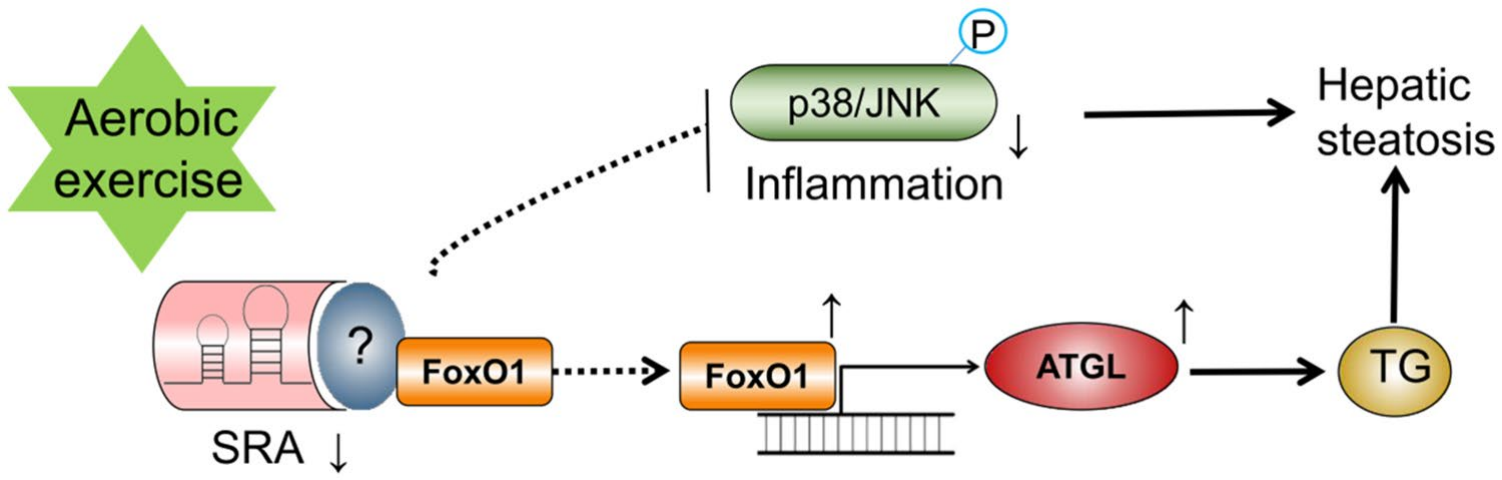
Aerobic exercise via SRA improves hepatic steatosis description model. Aerobic exercise is closely linked to the reduction of intrahepatic lipid aggregation and inflammatory responses through the inhibition of SRA levels. Mechanistically, aerobic exercise may inhibit the transcriptional activity of FoxO1 by suppressing SRA expression, which leads to upregulation of ATGL expression. In addition, remission of inflammation was accompanied by significant alteration of inflammatory proteins and the P38/JNK signaling pathway.

In summary, as shown in Fig. 5, we first demonstrated that aerobic exercise is closely linked to the reduction of intrahepatic lipid aggregation and inflammatory response through the inhibition of SRA levels, which are two keys to improving hepatic steatosis. Mechanistically, aerobic exercise may inhibit the transcriptional activity of FoxO1 by suppressing the expression of SRA, which leads to the upregulation of ATGL expression. In addition, remission of inflammation was accompanied by significant alteration of inflammatory proteins and P38/JNK signaling pathways. Our results suggest that SRA plays an important role in aerobic exercise to improve hepatic lipid metabolism.

## Supplementary Information

Supplementary Information.

## Abbreviations

NC: Normal control group
HFD: High-fat diet group
EV: Virus empty vector group
SRA + AE: SRA overexpression and combined with aerobic exercise group
TC: Total cholesterol
LDL-C: Low density lipoprotein cholesterin
P38: P38 protein
TNF-α: Tumor necrosis factor
IRE-1α: Inositol-requiring enzyme 1
NE: Normal aerobic exercise group
HAE: High-fat diet with aerobic exercise group
SRA+: SRA overexpression group
TG: Triglyceride
HDL-C: High density lipoprotein cholesterol
JNK: C-Jun *N*-terminal kinase
1L-6: Interleukin 6
1L-10: Interleukin 10
PERK: Protein kinase R-like endoplasmic reticulum kinase
