# Supplementary figures and images for: Aerobic exercise promotes the expression of ATGL and attenuates inflammation to improve hepatic steatosis via lncRNA SRA
Source: Sci Rep. 2022 Mar 30;12:5370. doi: 10.1038/s41598-022-09174-0 (PMC8968712; doi:10.1038/s41598-022-09174-0)

**The images of the original blots:**


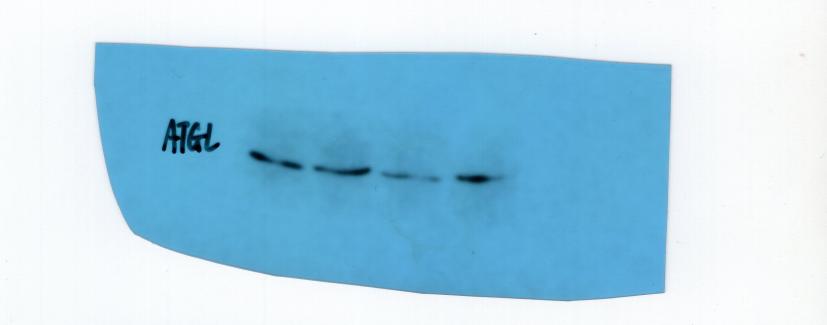
Figure 3.B


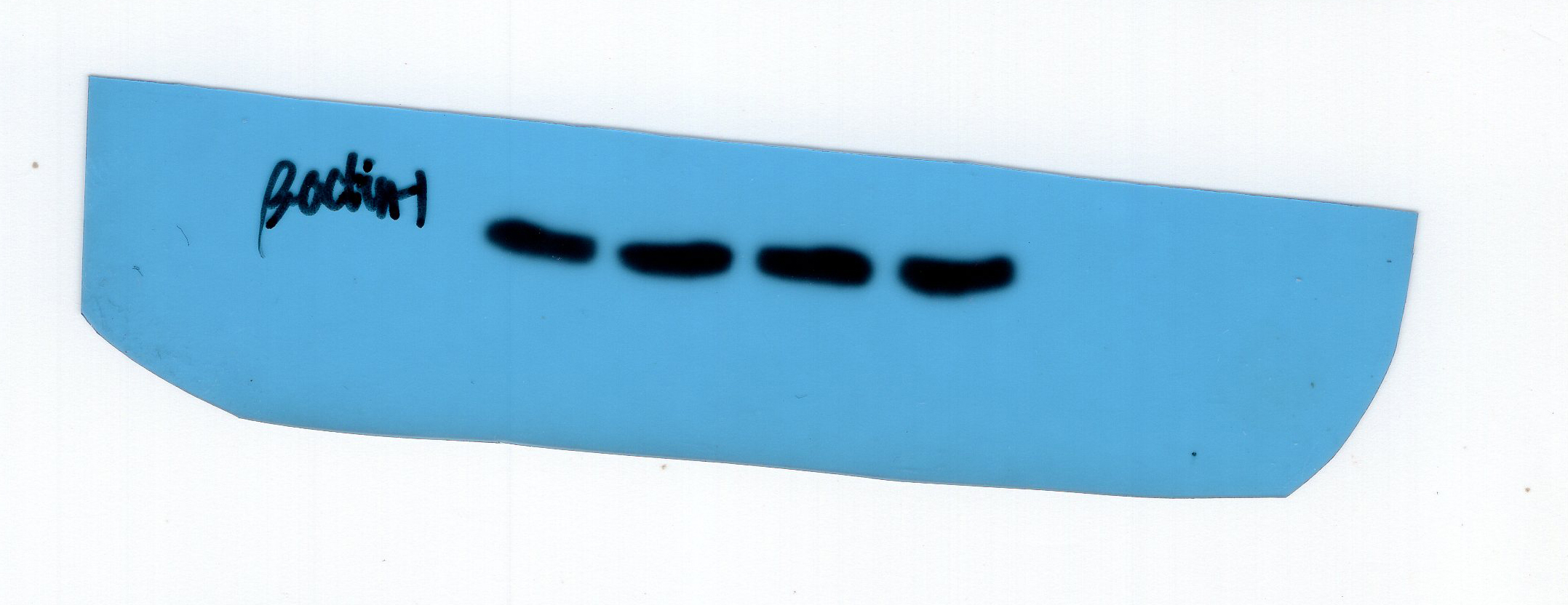


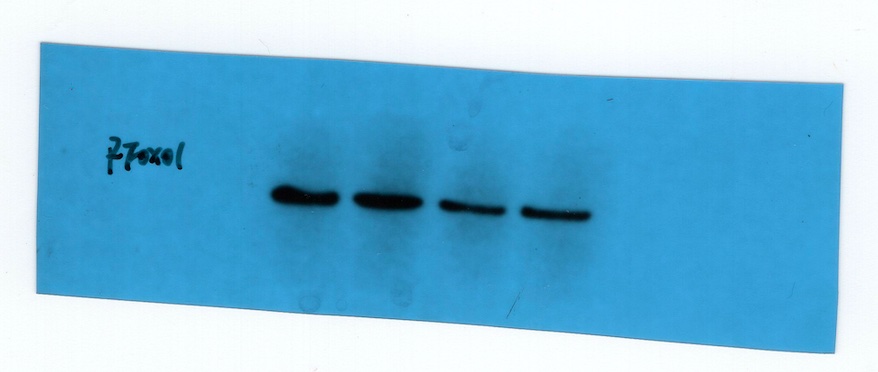


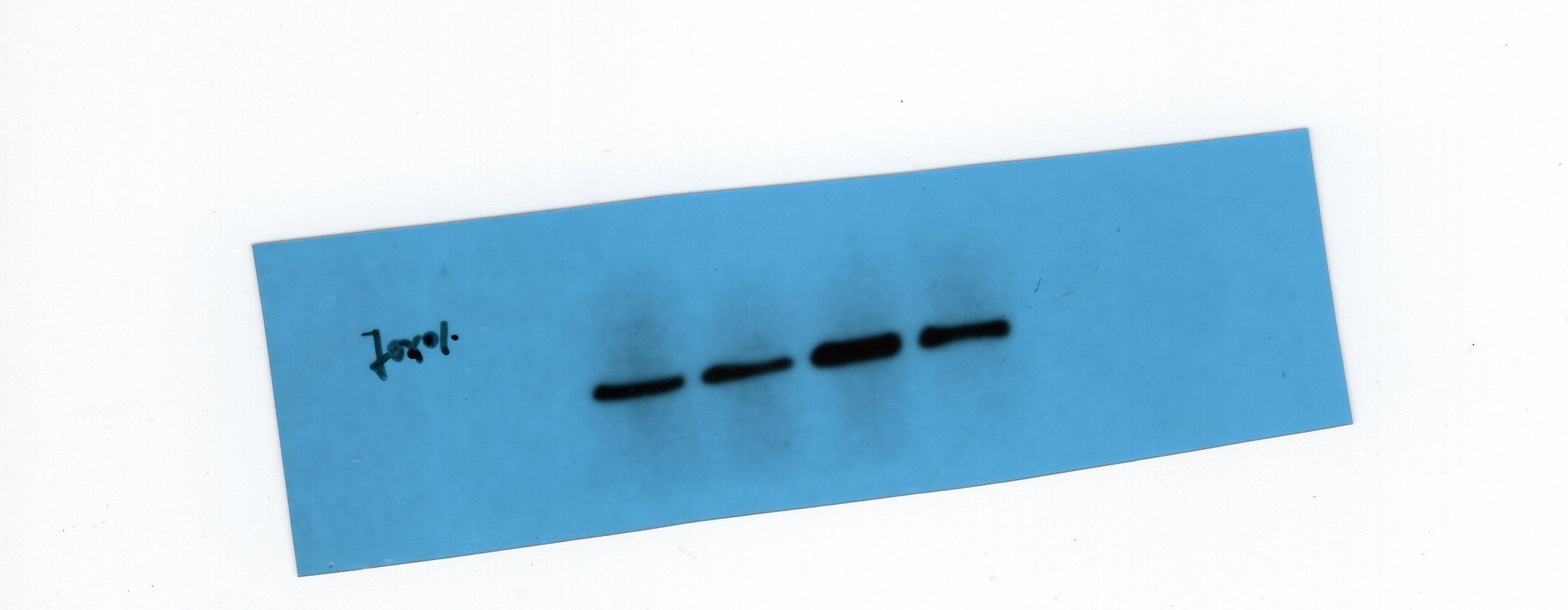
Figure 3.E


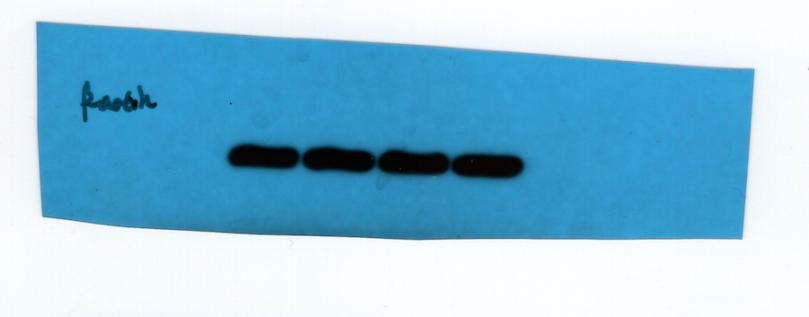


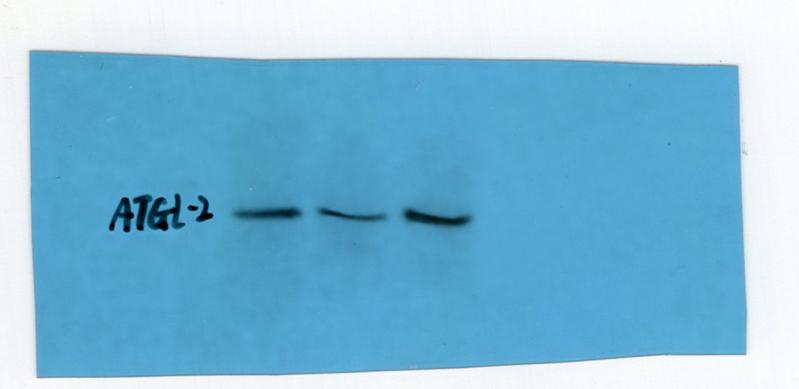


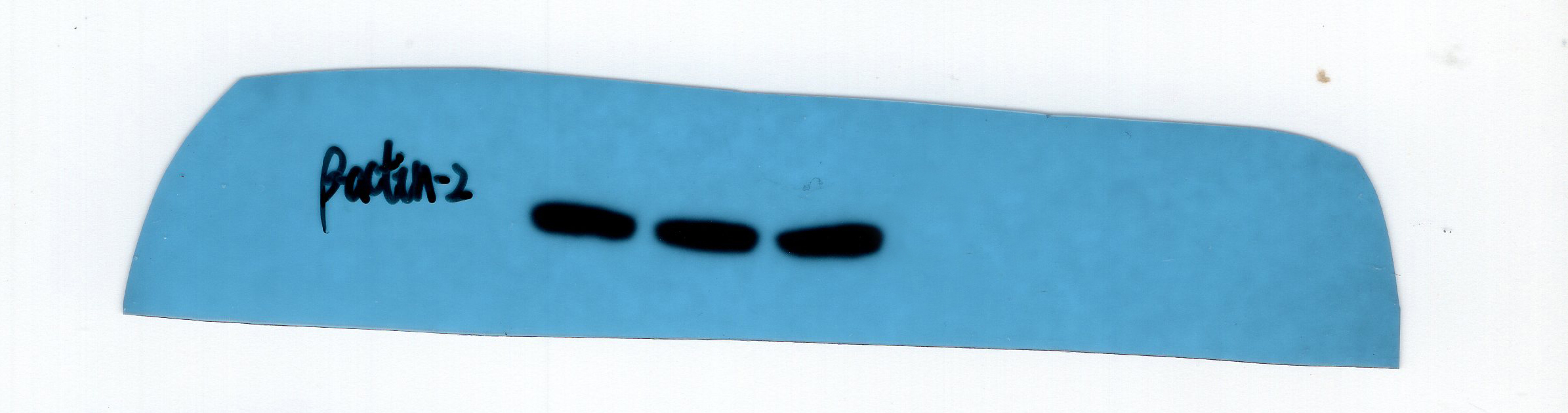
Figure 3.G

Figure 3.H


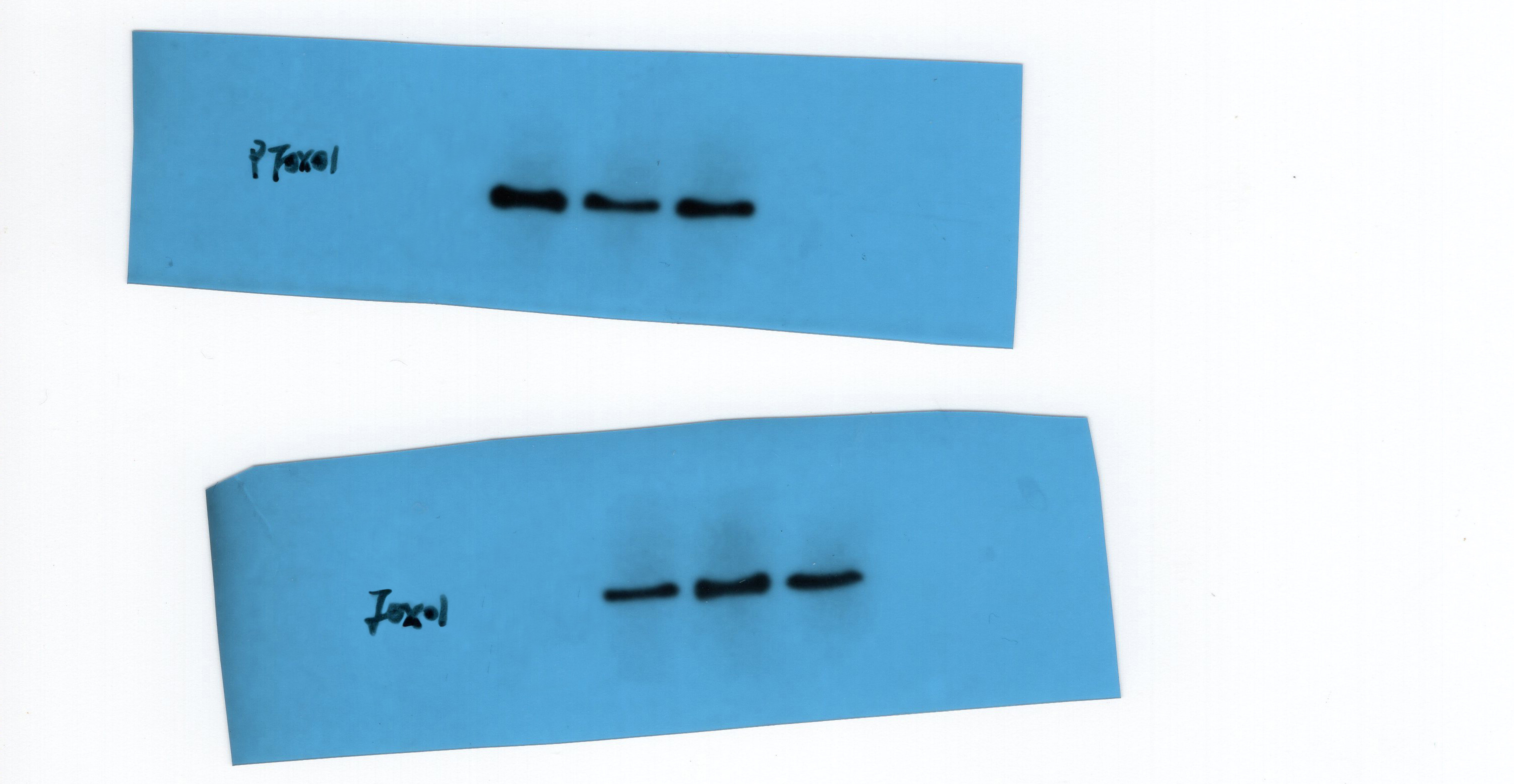


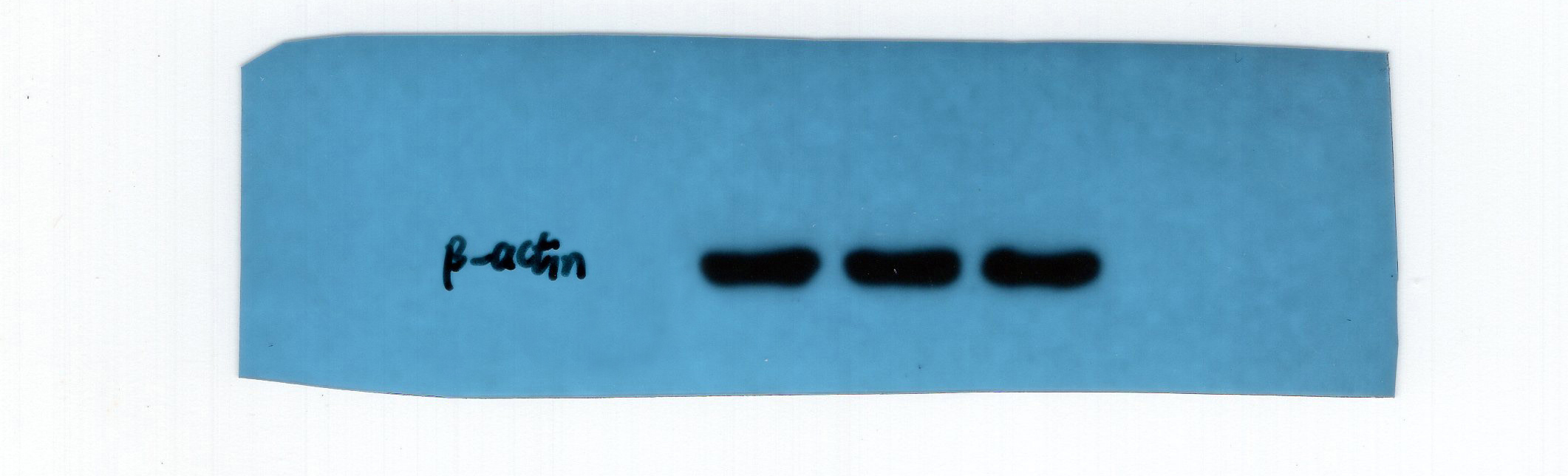


Figure 4.B


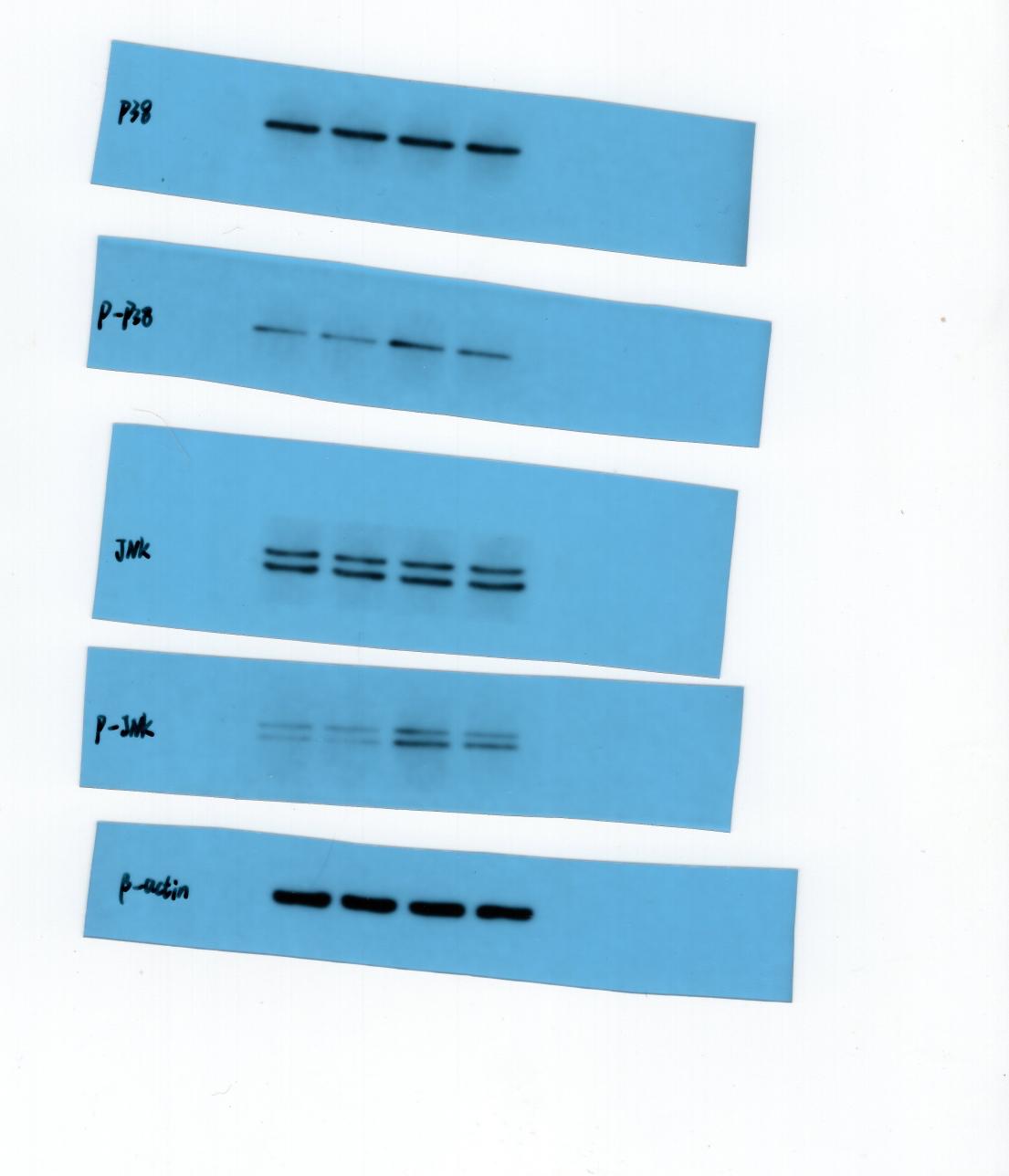


Figure 4.F


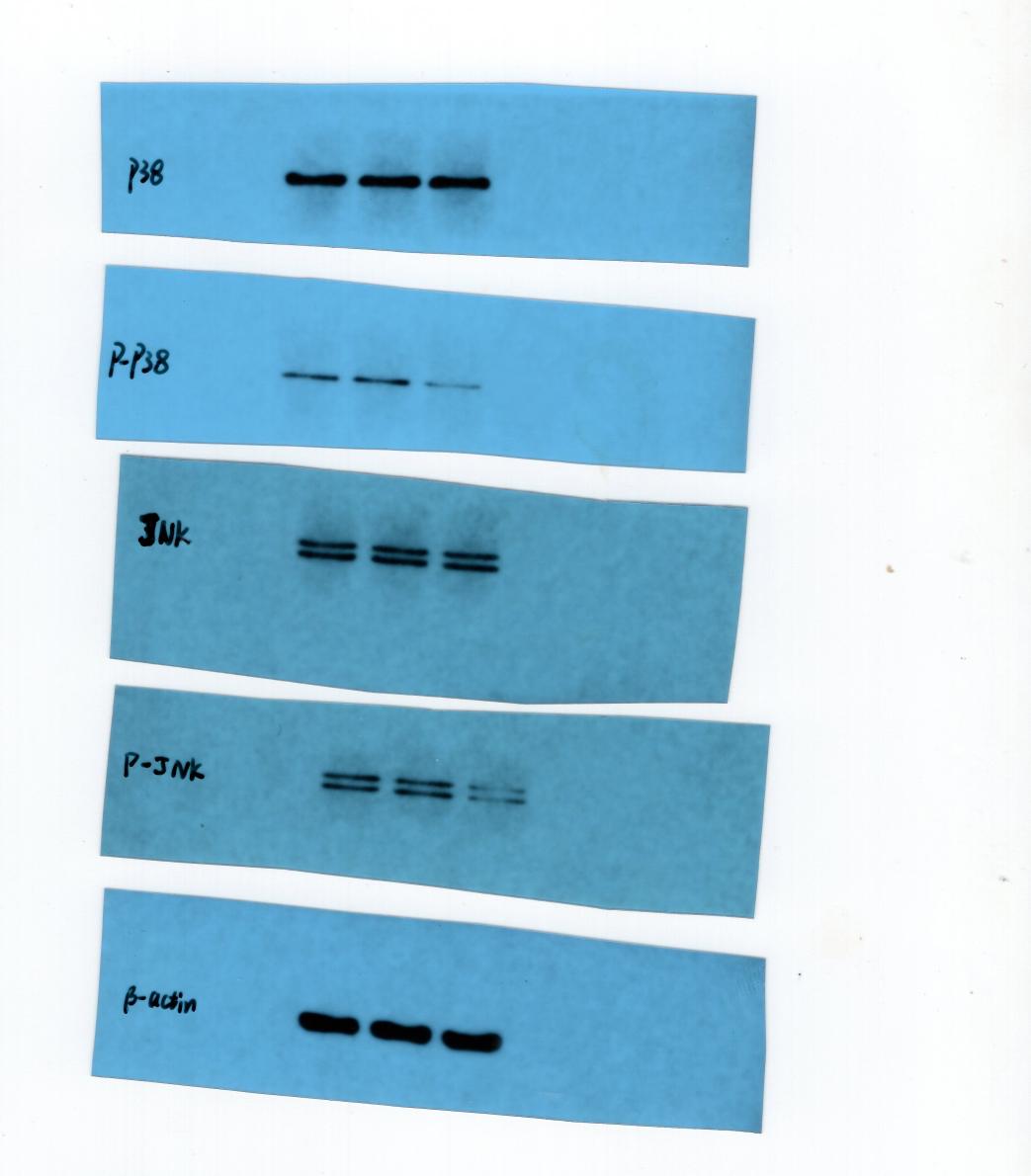

Supplement: Supplementary file 1 — Supplementary Information. [file 41598_2022_9174_MOESM1_ESM.docx]
